# Supplementary material for: Differential recognition of canonical NF-κB dimers by Importin α3
Source: Nat Commun. 2022 Mar 8;13:1207. doi: 10.1038/s41467-022-28846-z (PMC8904830; doi:10.1038/s41467-022-28846-z)
Supplement: Supplementary file 1 — Supplementary Information [file 41467_2022_28846_MOESM1_ESM.pdf]

Supplementary Information

Differential recognition of canonical NF-κB dimers by Importin α3

Tyler J. Florio [1], Ravi K. Lokareddy [1], Daniel P. Yeggoni [1], Rajeshwer S. Sankhala [2], Connor Ott [1], Richard E. Gillilan [3], and Gino Cingolani [1†]

[1] *Department of Biochemistry and Molecular Biology, Thomas Jefferson University, 1020 Locust Street, Philadelphia, PA 19107, USA*

[2] *Center of Infectious Disease Research, Walter Reed Army Institute of Research, Silver Spring, MD, USA*

[3] *Macromolecular Diffraction Facility, Cornell High Energy Synchrotron Source (MacCHESS), Cornell University, 161 Synchrotron Drive, Ithaca, NY 14853, USA*

[†] Corresponding author: E-mail: gino.cingolani@jefferson.edu Tel.: (215) 503 4573; FAX: (215) 464 4595;

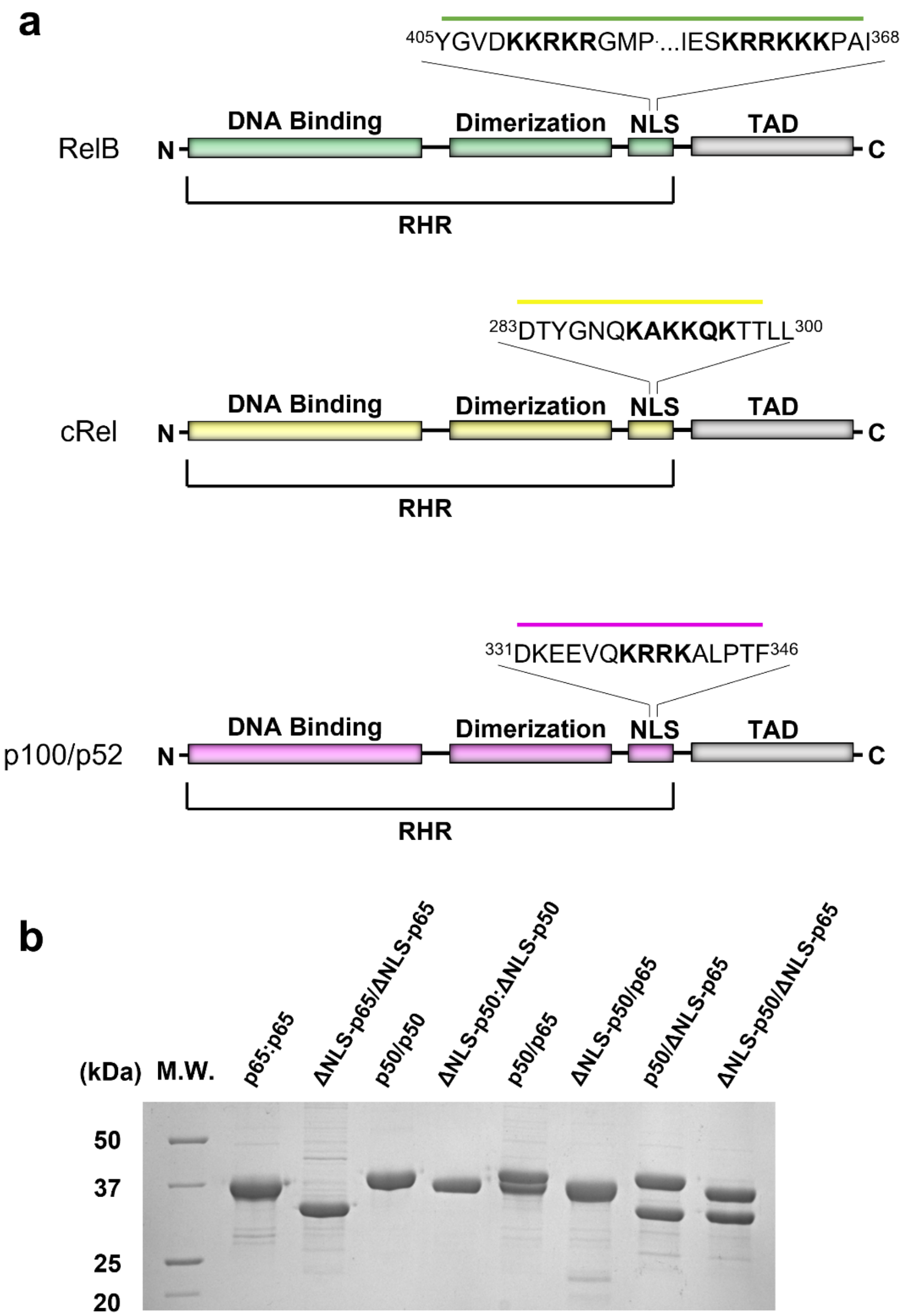

Supplementary Figure 1. Schematic diagram of NF-κB subunits and purification of NF-κB complexes. (**a**) Schematic diagram of RelB, cRel, and p100/p52 subunits, highlighting the position and sequence of the functional NLSs. (**b**) SDS-PAGE analysis of all purified NF-κB homo- and heterodimers used in this study. All NF-κB homodimers (e.g., p50/50, p65/p65, ΔNLS-p50/ΔNLS-p50, and ΔNLS-p65/ΔNLS-p65) were formed in bacteria, while all heterodimers (e.g., p50/p65, ΔNLS-p50/p65, p50/ΔNLS-p65, and

ΔNLS-p50:ΔNLS-p65) were assembled *in vitro* from purified components. Source data are provided as a Source Data file.

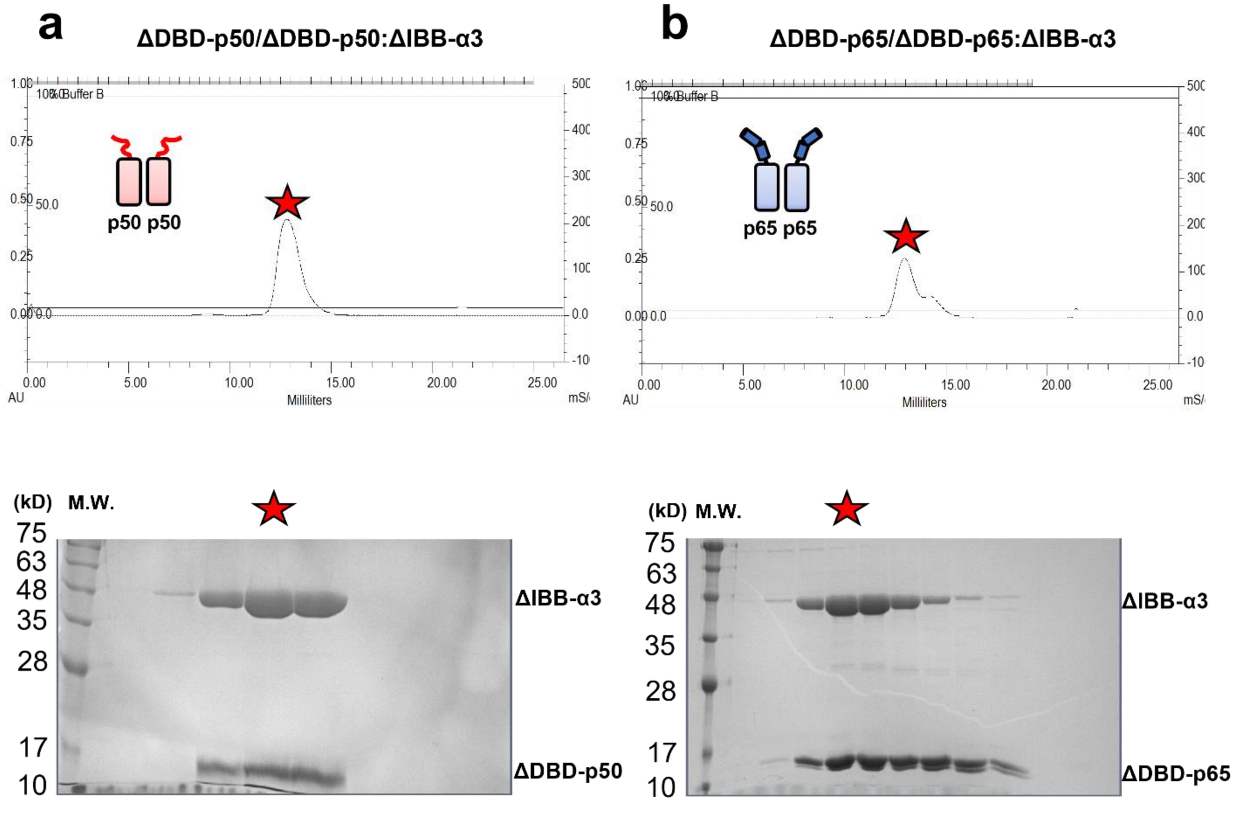

Supplementary Figure 2. SEC analysis of NF-κB dimerization domains (ΔDBD-p65/ ΔDBD-p65 and ΔDBD-p50/ΔDBD-p50) co-expressed in bacteria with ΔIBB-importin α3. *Top panels:* the ΔDBD-p50/ΔDBD-p50:ΔIBB-importin α3 trimeric complex eluted as a single peak (**a**), while the ΔDBD-p65/ΔDBD-p65:ΔIBB-importin α3 complex had a shoulder, corresponding to free ΔDBD-p65 homodimer (**b**). *Bottom panels:* SDS-PAGE analysis of peak fractions (denoted by a red star) confirming the smaller peak in (**b**) contains an excess of free ΔDBD-p65. The data shown in panels **a** and **b** are representative of two independent experiments. Source data are provided as a Source Data file.

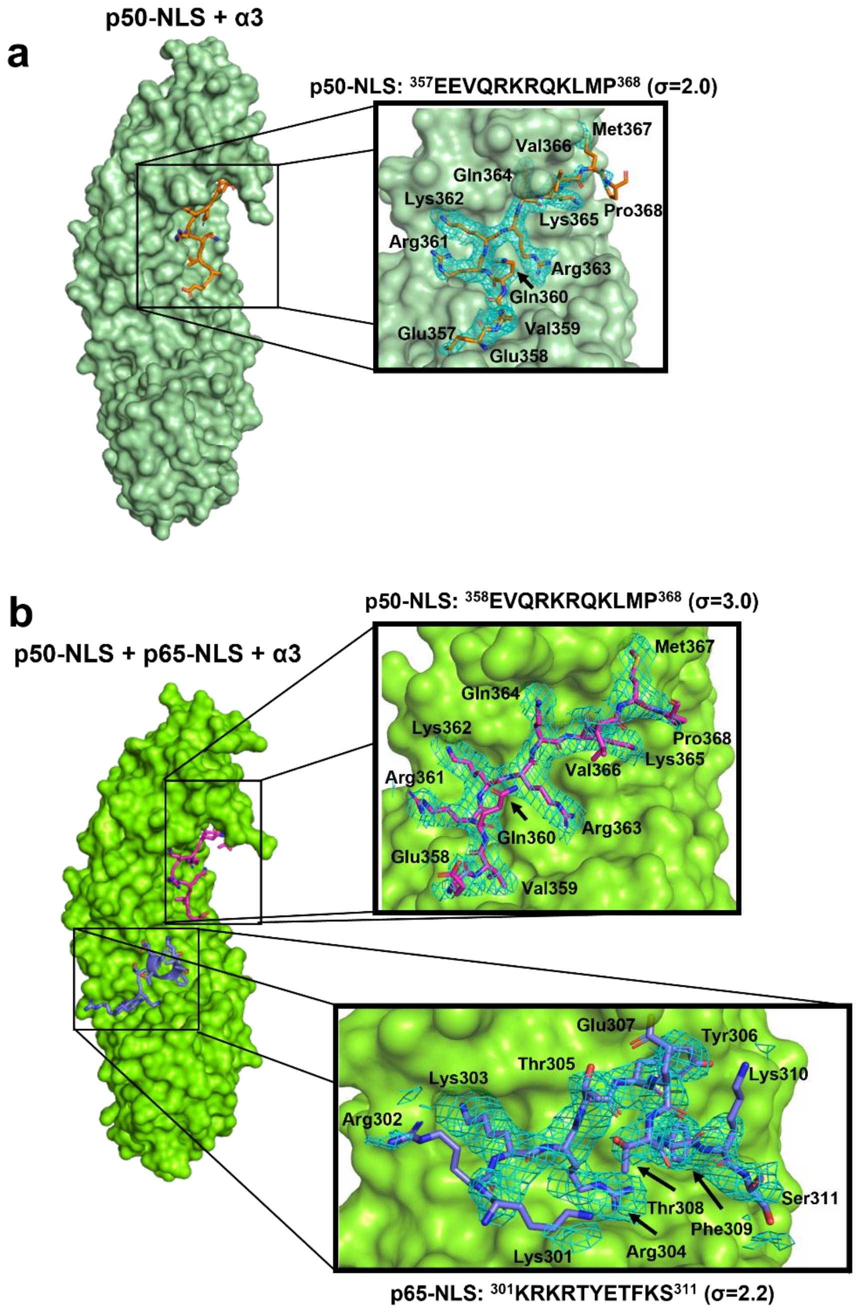

Supplementary Figure 3. Electron density maps of NF-κB NLS peptides bound to ΔIBB-importin α3. The Zoom-in panels show the Polder [1] electron densities for the NF-κB NLSs (colored in cyan) calculated after omitting the peptides from the final models: (**a**) p50-NLS crystallized alone (orange) with ΔIBB-importin α3; (**b**) p50-NLS + p65-NLS (colored in magenta and blue, respectively) co-crystallized with ΔIBB-importin α3. All Polder densities were contoured between 2-3 σ above the background, as indicated in each panel. The ΔIBB-importin α3 is shown as a solvent surface colored in two shades of green, as in **Fig. 4a,b**.

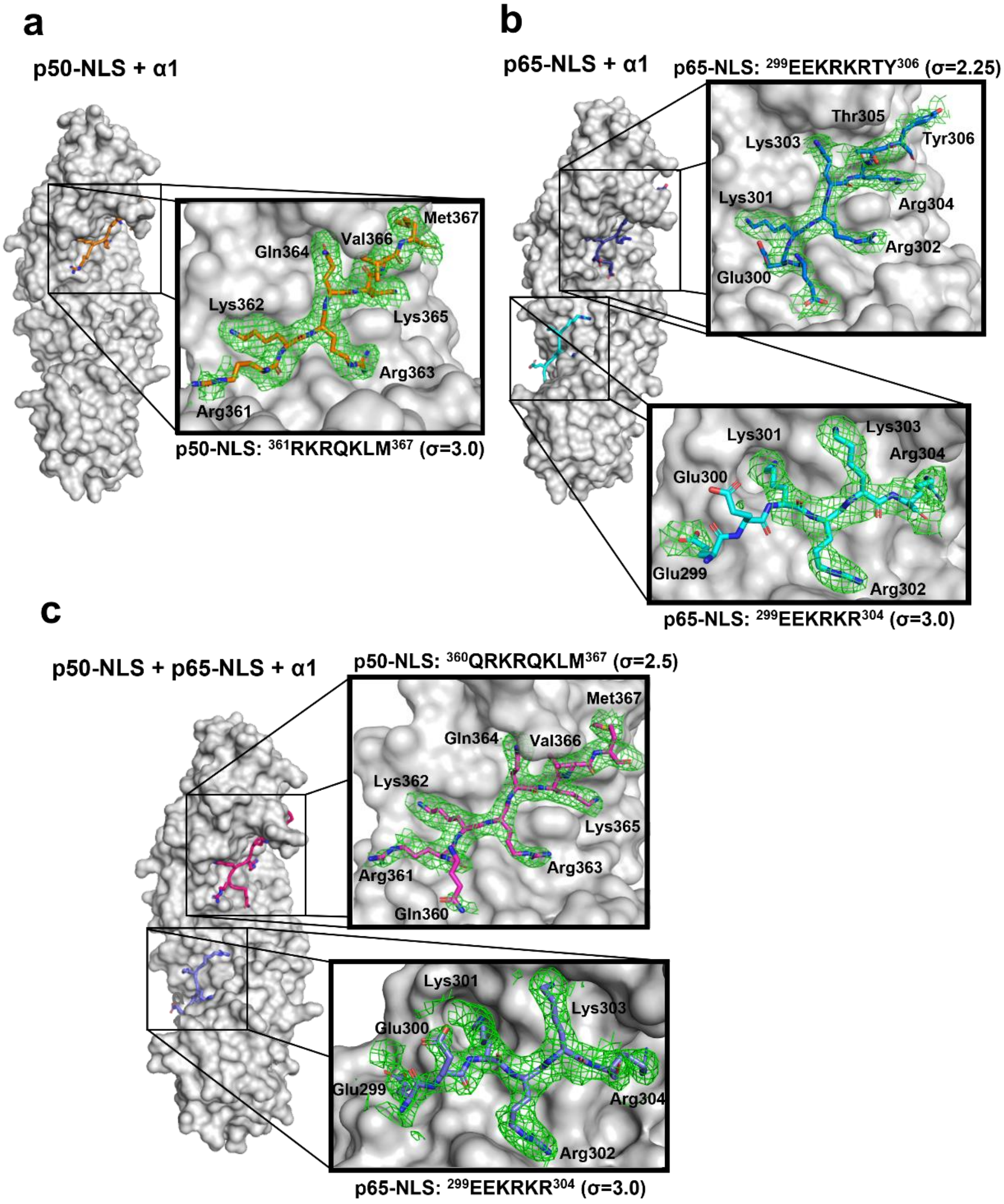

Supplementary Figure 4. Electron density maps of NF-κB NLS peptides bound to ΔIBB-importin α1. The Zoom-in panels show the Polder [1] electron densities for the NF-κB NLSs (colored in green) calculated after omitting the peptides from the final model: (**a**) p50-NLS crystallized alone (orange) with ΔIBB-importin α1; (**b**) p65-NLS crystallized with ΔIBB-importin α1 (colored in dark blue and cyan), and (**c**) p50-NLS + p65-NLS (colored in magenta and blue, respectively) co-crystallized with ΔIBB-importin α1. All Polder densities were contoured between 2-3 σ above the background, as indicated in each panel. In all panels, ΔIBB-importin α1 is shown as a solvent surface colored in gray.

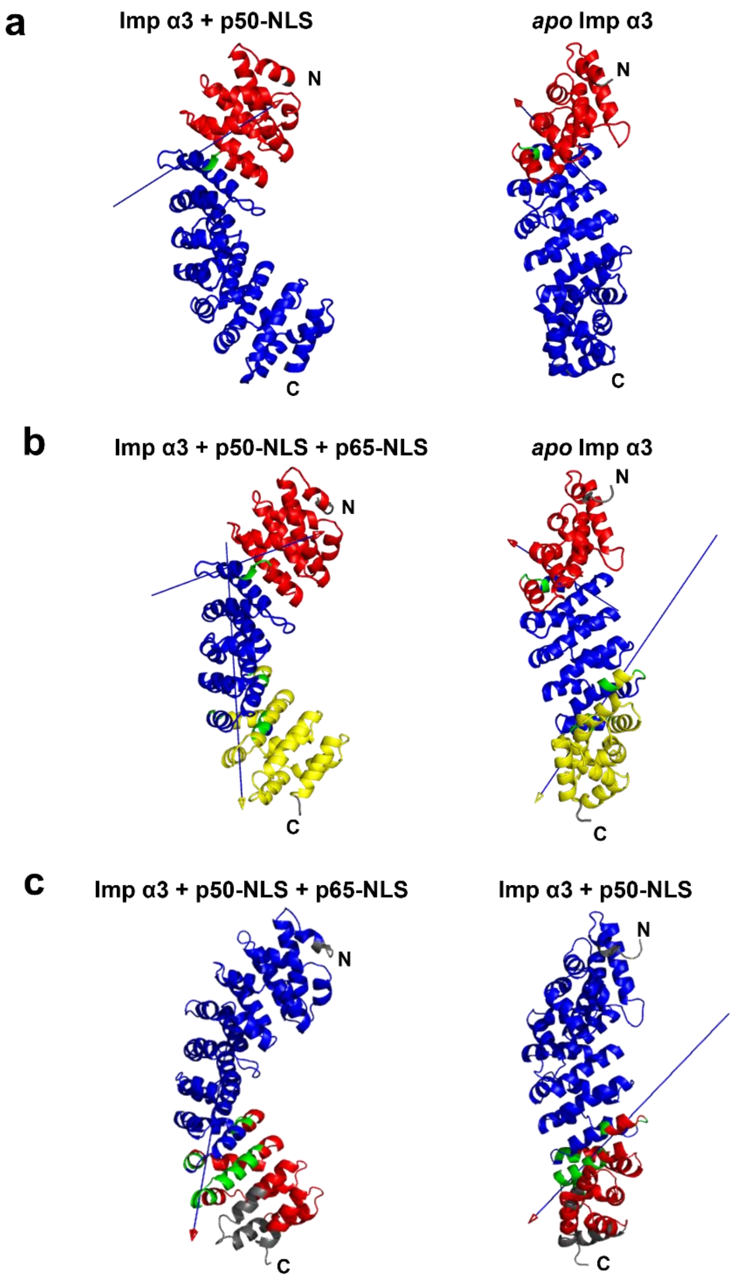

Supplementary Figure 5. DynDom analysis of importin α3 conformational plasticity in response to NF-κB NLS-binding. DynDom analysis comparing importin α3 conformational changes between (**a**) p50 NLS-bound (PDB: 7LFC) and *apo* importin α3 (PDB: 6BVZ), (**b**) p50/p65 NLS-bound (PDB: 7LF4 Chain C) and *apo* importin α3, and (**c**) p50/p65 NLS-bound and p50 NLS-bound importin α3. Domains 1, 2, and 3 are colored in red, blue, and yellow, respectively. Arms undergoing conformational changes are colored red and yellow, whereas residues around which motion occurs in green. Regions poorly defined by DynDom [2] analysis are gray. A blue line with a red or yellow arrowhead shows the location of the conformational changes.

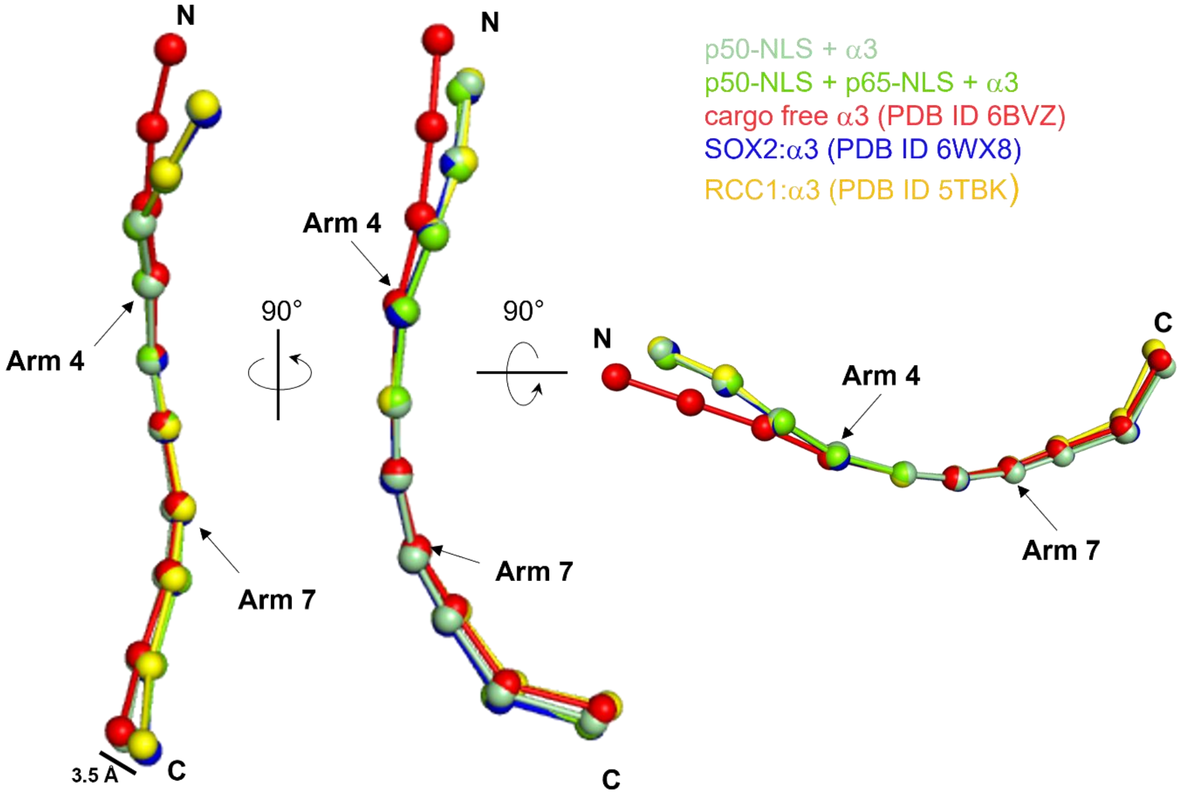

Supplementary Figure 6. Comparison of all importin α3 crystal structures.

Residues 72-486 of importin α3 (shown as a beads-on-a-string) in the cargo-free conformation (red) (PDB: 6BVZ) or bound to p50-NLS (light green) (PDB: 7LFC), p50-NLS + p65-NLS (green) (PDB: 7LF4 Chain C), SOX2 (blue) (PDB: 6WX8), and RCC1 (yellow) (PDB: 5TBK) cargo were superimposed using SuperPose [3]. Beads-on-a-string models were drawn in PyMoL (Schrödinger, Inc.) and are based upon the conserved tryptophan α-carbon in the center of third Arm helices, as previously described [4].

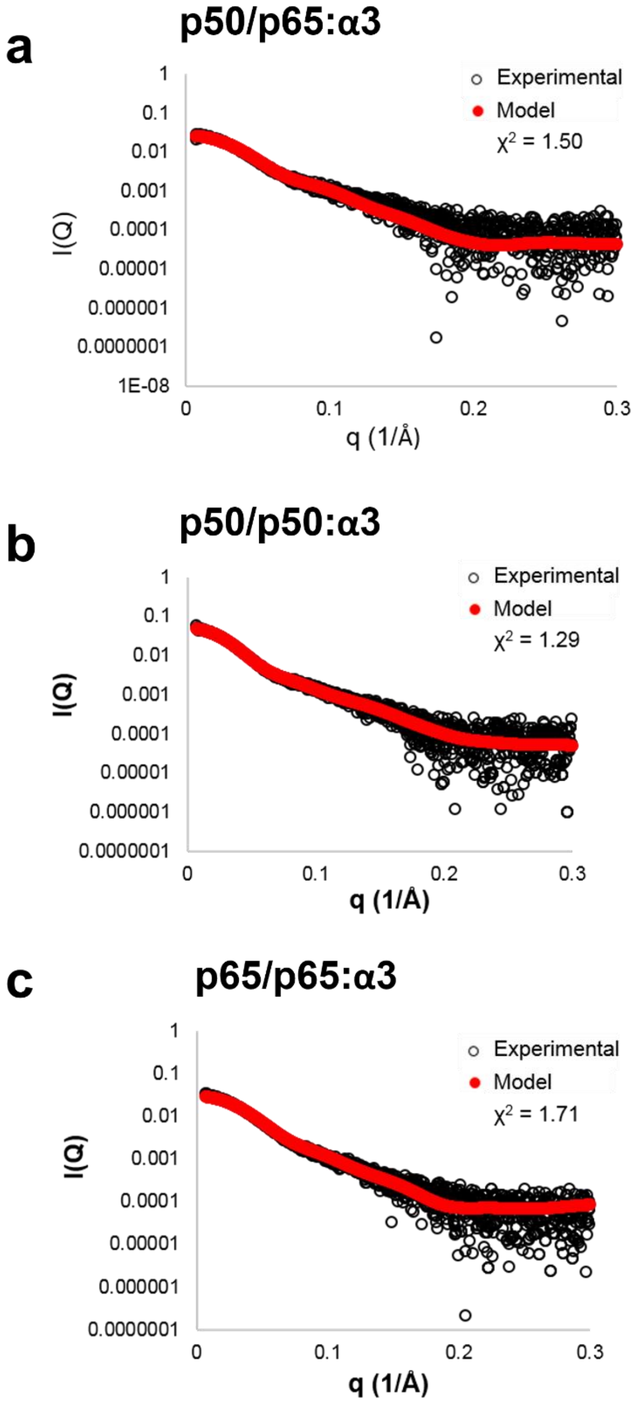

Supplementary Figure 7. NF-κB:ΔIBB-importin α3 SEC-SAXS analysis and agreement. Overlay of the experimental SEC-SAXS data (black circles) and scattering data (red line) calculated from the DENSS model of p50/p65:ΔIBB-Importin α3 (**a**), p50/p50:ΔIBB-Importin α3 (**b**), and p65/p65:ΔIBB-Importin α3 (**c**). The χ^2 values were calculated using the FoXS server [5]. Source data are provided as a Source Data file.

Supplementary Table 1: NF-κB/Importin α3 Complex Molecular Weights

Importin **α**	NF-**κ**B	**Cumulative MW**
His-ΔIBB-Importin α3		~52.6 kDa
ΔIBB-Importin α3		~46.4 kDa
	p50/p50	~80 kDa
	p65/p65	~72.8 kDa
	p50/p65	~76.4 kDa
	ΔDBD-p50/p65	~50.8 kDa
	ΔDBD-p50/ΔDBD-p50	~28.8 kDa
	ΔDBD-p65/ΔDBD-p65	~29.2 kDa
His-ΔIBB-Importin α3	p50/p65	~129 kDa
His-ΔIBB-Importin α3	p50/p50	~185.2 kDa
His-ΔIBB-Importin α3	p65/p65	~125.4 kDa
ΔIBB-Importin α3	ΔDBD-p50/p65	~97.2 kDa
ΔIBB-Importin α3	ΔDBD-p50/ΔDBD-p50	~75.2 kDa
ΔIBB-Importin α3	ΔDBD-p65/ΔDBD-p65	~75.6 kDa

Supplementary Table 2. Structural alignment of importin α/Kap60 solved in complex with NLS.

NLS type	Minor Binding Site<br>P_{-1}' P_0' P_1' P_2' P_3' P_4' P_5'		Major Binding Site<br>P_0 P_1 P_2 P_3 P_4 P_5	PDB ID
**SV40T-ag**	P K K **K** R K		P K **K** K R K V	1EJL/1BK6*
**hPLSCR1-NLS**			G **K** I S K HWTGI	1Y2A
**hPLSCR4-NLS**	S I I **R** K W N			3Q5U
**Ku70**		NEGS	G S **K** R P K VG	3RZX
**Ku80**		DGPT	T A **K** K L K TEQ	3RZ9
**B54-NLS**	L G K **R** K R H			2YNS**
**TPX2**	K **R** K H		P V **K** M I K	3KND
**C-Myc**	K **R** V K L		P A A **K** R V K	1EE4**
**Nucleoplasmin**	A V K **R** P A A	TKKAG	Q A K **K** K K L D	1EJY/1EE5*
**Kap60-IBB**	E L R **R** R R D	TQQVELRKAKRDEA	L A **K** R R N F	1WA5*
**h1NLS**	TR K K **R** K D P	DSDDWSES	N S **K** E N K ID	4XZR *
**h2NLS**	T N K **R** K R E	QISTDNEAKMQIQEEKS	P K **K** K R K KRSSKANK	4PVZ*
**NP-NLS**	SQGT K **R** S Y E QM			4ZDU
**p50-NLS[1]**			Q R **K** R Q K LM	7LEQ/7LET
**p50-NLS[2]**		EV	Q R **K** R Q K LMP	7LFC/7LF4
**p65-NLS[1]**	E K **R** K R		E **K** R K R	7LEU
**p65-NLS[1]**	E E K **R** K R			7LET
**p65-NLS[2]**	K R K **R** T Y E TFKS			7LF4

* denotes yeast importin α (Kap60).
** denotes yeast importin α (Kapα50)
[1] bound to importin α1
[2] bound to importin α3

Supplementary Table 3. SEC-SAXS data collection and refinement statistics

**SEC-SAXS**	**p50/p65:∆IBB-Imp α3**	**p50/p50:∆IBB-Imp α3**	**p65/p65:∆IBB-Imp α3**
Instrument	ID7A1	ID7A1	ID7A1
Wavelength (Å)	1.237	1.237	1.237
Exposure time (s)	2.0	2.0	2.0
Protein Concentration (mg ml^{-1})	4.5	4.5	4.5
Temperature (oK)	277	277	277
Radius of Gyration, R_g* (Å)	44.21 $\pm$ 0.28	48.56 $\pm$ 0.22	44.50 $\pm$ 0.24
Maximum Diameter, D_{max}	137	157	151
Volume of Correlation M.W. / Theoretical M.W. (kDa)	124.4 $\pm$ 12.4 / 129	200.7 $\pm$ 20.1 / 185.2	146 $\pm$ 14.6 / 125.4
	**Software employed**		
Primary Data Reduction	RAW version 1.6.4	RAW version 2.0.3	RAW version 2.0.3
Data Processing	ATSAS	ATSAS	ATSAS
Ab initio analysis	DENSS	DENSS	DENSS
Validation and averaging	EMAN2	EMAN2	EMAN2
Rigid-body refinement	*Phenix.real_space_refine*	*Phenix.real_space_refine*	*Phenix.real_space_refine*
Computation of model intensities	FoxS	FoxS	FoxS
3D-graphics representations	Chimera	Chimera	Chimera

* Rg was determined from the Guinier plot.

Supplementary References

1 Liebschner, D. *et al.* Polder maps: improving OMIT maps by excluding bulk solvent. *Acta Crystallogr D Struct Biol* **73**, 148-157, doi:10.1107/S2059798316018210 (2017).

2 Hayward, S. & Berendsen, H. J. Systematic analysis of domain motions in proteins from conformational change: new results on citrate synthase and T4 lysozyme. *Proteins* **30**, 144-154, doi:10.1002/(SICI)1097-0134(19980201)30:2<144::AID-PROT4>3.0.CO;2-N [pii] (1998).

3 Maiti, R., Van Domselaar, G. H., Zhang, H. & Wishart, D. S. SuperPose: a simple server for sophisticated structural superposition. *Nucleic Acids Res* **32**, W590-594 , doi:10.1093/nar/gkh477 [pii] (2004).

4 Pumroy, R. A., Ke, S., Hart, D. J., Zachariae, U. & Cingolani, G. Molecular determinants for nuclear import of influenza A PB2 by importin alpha isoforms 3 and 7. *Structure* **23**, 374-384, doi:10.1016/j.str.2014.11.015 [pii] (2015).

5 Schneidman-Duhovny, D., Hammel, M. & Sali, A. FoXS: a web server for rapid computation and fitting of SAXS profiles. *Nucleic Acids Res* **38**, W540-544, doi:10.1093/nar/gkq461 [pii] (2010).
